# Treatment of splenic trauma in Norway: a retrospective cohort study

**DOI:** 10.1186/s13049-017-0457-y

**Published:** 2017-11-23

**Authors:** Trond Dehli, Jorunn Skattum, Bjørn Christensen, Ole-Petter Vinjevoll, Bent-Åge Rolandsen, Christine Gaarder, Pål Aksel Næss, Torben Wisborg

**Affiliations:** 1 Department of Gastrointestinal Surgery, University Hospital North Norway Tromsø, Tromsø, Norway; 2 Anaesthesia and Critical Care Research Group, Faculty of Health Sciences, University of Tromsø, Tromsø, Norway; 3 Department of Traumatology, Division of Emergencies and Critical Care, Oslo University Hospital Ullevål, Oslo, Norway; 4 Emergency Care Clinic, Haukeland University Hospital, Bergen, Norway; 5 Department of Surgery, St. Olav’s University Hospital, Trondheim, Norway; 6 Fauculty of Medicine, University of Oslo, Oslo, Norway; 7 Norwegian National Advisory Unit on Trauma, Division of Emergencies and Critical Care, Oslo University Hospital Ullevål, Oslo, Norway

**Keywords:** Splenic injury, Nonoperative management, Splenic artery embolization, Incidence

## Abstract

**Background:**

Non-operative management of splenic injuries has become the treatment of choice in hemodynamically stable patients over the last decades. The aim of the study is to describe the incidence, initial treatment and early outcome of patients with splenic injuries on a national level.

**Methods:**

All hospitals in Norway admitting trauma patients were invited to participate in the study. The study period was January through December 2013. The hospitals delivered anonymous data on primarily admitted patients with splenic injury.

**Results:**

Three of the four regional trauma centers and 26 of the remaining 33 acute care hospitals delivered data on a total of 151 patients with splenic injury indicating an incidence of 4 splenic injuries per 100,000 inhabitants/year, and a median of 4 splenic injuries per hospital per year. A total of 128 (85%) patients were successfully treated non-operatively including 20 patients who underwent an angiographic procedure. The remaining 23 (15%) patients underwent open splenectomy or spleen-preserving surgery.

**Conclusion:**

Most patients with splenic injuries are managed non-operatively. Despite the low number of splenic injuries per hospital, the results indicate satisfactory outcome on a national level.

## Background

The management of blunt splenic injuries has evolved significantly over the years [1]. Historically, splenectomy was the treatment of choice and still is in the exsanguinating patient where the spleen is a significant source of bleeding. The recognition of the immunological function of the spleen and the laparotomy-associated morbidity has led to the shift from operative management (OM) to non-operative management (NOM) in the approximately 70% of adult patients who are hemodynamically stable on hospital admission [1, 2]. Over the past 20 years, success rates of NOM in this category of patients have continued to improve, with rates greater than 90% in most trauma centers when splenic artery embolization (SAE) is included as part of the treatment algorithm [1–4].

In Norway, Oslo University Hospital Ullevål (OUHU) introduced SAE as a part of a treatment algorithm for splenic injury in 2002 [5]. The current algorithm mandates SAE in all hemodynamically normal or normalized patients with Organ Injury Scale (OIS) grade 4 and 5 splenic injuries [6], as well as in stable patients with contrast extravasation seen on computed tomography (CT) scan regardless of injury grade [2]. The resulting NOM success rate at OUHU is comparable with those reported from the US both in children and adults with a success rate of >90% [1–4, 7]. Angiographic service has later been established in the other three trauma centers and eight acute care hospitals. The outcome of splenic injury treatment at the University Hospital North Norway Tromsø is similar to OUHU [8], but results on a national level remain unknown.

The Norwegian national organization of trauma care has been developed through two national expert white papers, which have subsequently been adopted by the regional health trusts [9, 10]. The white papers mandate the availability of an interventional radiology service in each trauma centre, but do not include national guidelines specifying the management of splenic injury. Traumatic splenic injury is a well-defined entity, and in a rather homogenous population the care and outcome may reflect system results on a national level [11, 12].

On that background, the present study aimed at describing the incidence, initial treatment in the first admitting hospital and early outcome in patients with splenic injuries.

## Methods

The mainland of Norway covers an area of 385,000 km^2^ and is populated by 5 million inhabitants. There were 37 hospitals admitting trauma patients at the time of the study. The Norwegian trauma system consists of four independent Regional Health Trusts, with two levels of admitting hospitals. Each region has one trauma center, and between five and 15 acute care hospitals. The regional trauma centers have all medical capabilities, similar to the level I and II trauma centers described by the American College of Surgeons, Committee on Trauma (ACS-COT) [9, 13]. The acute care hospitals are similar to the ACS-COT level III centers and are capable of managing the majority of injured patients and stabilizing severely injured patients before transfer to the trauma centers when indicated according to transfer criteria. For stabilized patients with splenic injuries primarily admitted to an acute care hospital, transfer to the regional trauma center is an option for continuous treatment with NOM with or without SAE [8]. Norway has an extensive prehospital ambulance service including air ambulance, which covers parts of both primary admissions and transfers between hospitals [14].

The study is a retrospective observational cohort study.

All patients with splenic injury (discharge diagnosis (ICD-10) S36.0) [15] primarily admitted in a hospital in Norway during the period of 1. Jan 2013–31. Dec 2013 were eligible for inclusion. Patients with no splenic injury (coding error) or iatrogenic splenic injury (perioperative injury) were excluded.

The study population is described with gender, age, mechanism of injury, classification of the splenic injury with OIS and Injury Severity Score (ISS) [16, 17]. The treatment of splenic injuries was categorized as spleen related OM (spleen preserving procedures or splenectomy) and NOM with observation only or NOM with SAE. NOM is considered successful if splenectomy or spleen-preserving surgery is avoided. Moreover, data registered include length of stay, transfusions, transfer to the regional trauma center, recorded complications to the treatment given at the primary facility, and mortality.

Data are presented using frequencies and medians with Inter Quartile Range (IQR).

The Regional Ethics Committee, REK Nord, waived the need for board review (case number 2014/1092/REK Nord). The patient data protection officers at all hospitals contributing with data gave permission to the study (Case number for the responsible hospital UNN Tromsø: 2015/3388).

## Results

**Fig. 1:**
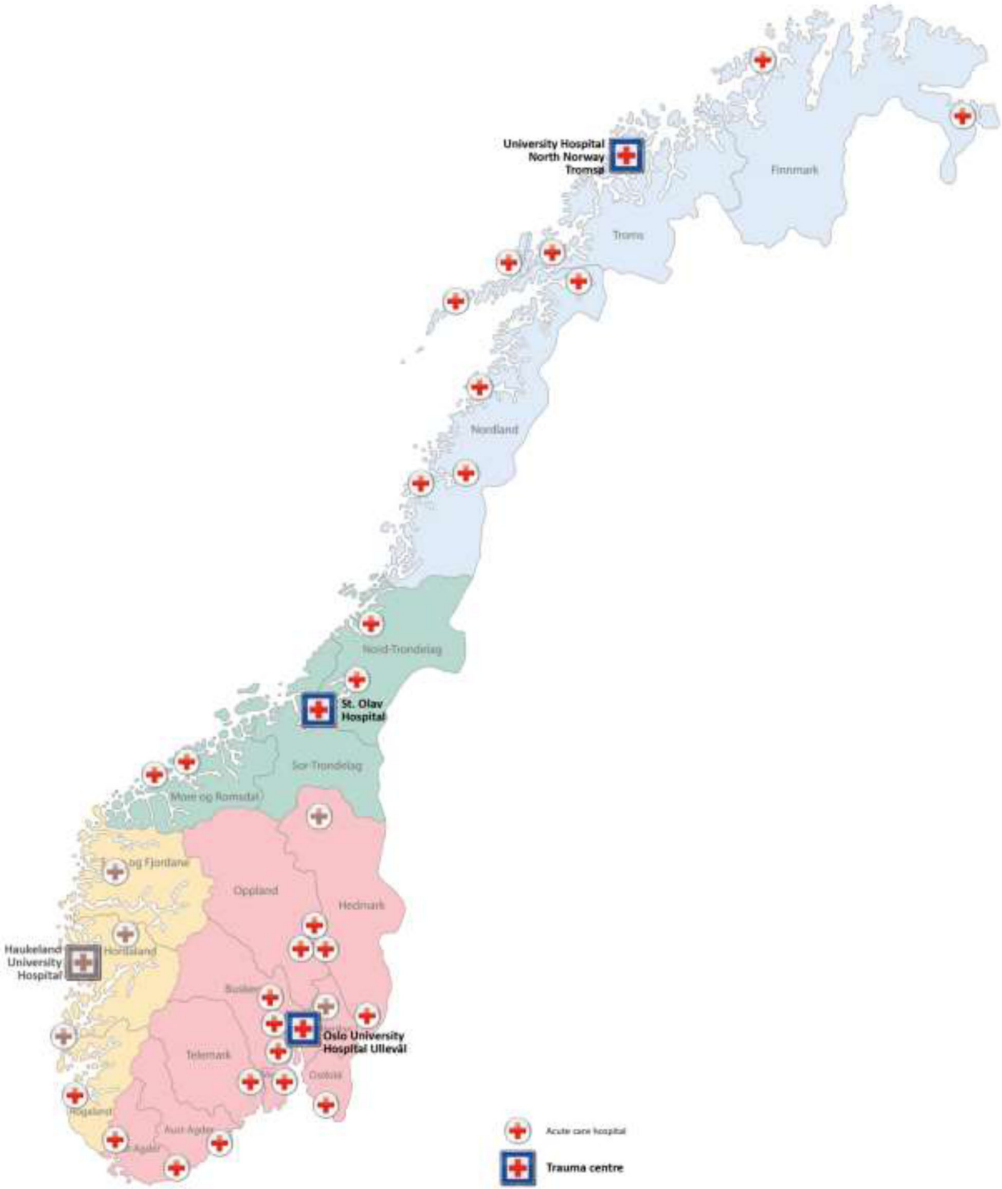
The four regional health trusts in Norway with all acute care hospitals and trauma centers. Hospitals with brown colours did not participate in the study

A total of 151 patients were included in the study. Three of the four trauma centers and 26 of the 33 acute care hospitals contributed data (Fig. 1). In four hospitals, the patient data protection officer did not approve the study, and four hospitals did not respond to repeated requests for data. OUHU was the largest contributor to the study with 36 included patients; the second largest contributor was Sørlandet Sykehus Kristiansand with 13 included patients. With 0–36 admitted patients, the median number of patients treated per hospital/year was 4.

The contributing hospitals cover a population of approximately 3.5 million people. With 151 patients admitted with splenic injury in these hospitals, the incidence of splenic injury was 4.3 per 100,000 inhabitants/year.

In Table 1 outcome data are presented for all patients and stratified for splenic injury grade. Of the 102 men and 49 women included in the study, the median (IQR) age was 28 (15, 50) years, and 39 (26%) patients were <16 years of age. The mechanism of injury was blunt in 96% secondary to the following four most frequent categories: road traffic related (52%), low energy falls (18%), high energy falls (17%) and hit by a blunt object (6%). In this study population, 128 (85%) patients were treated non-operatively. However, one patient treated with observation showed signs of delayed bleeding and underwent successful SAE. Splenectomy rate increased from 2% in OIS grade 1 and 2 injuries, to 15% in grade 3, and finally 30% in grade 4 and 5 injuries. Among the children, 34 of 39 were treated successfully with NOM including angiography in two; four children underwent splenectomy and one child underwent spleen-preserving surgery. A total of 6 (4%) patients died within 30 days after injury.

**Table 1:** Characteristics for 151 patients stratified by splenic injury grade

	All patients	OIS 1&2	OIS 3	OIS 4&5
Patients, n	151^a^	50	53	47
ISS, median (IQR)	16 (9,24)	13 (6,24)	13 (9,22)	17 (16,25)
Transfusion ≥1 unit RBC, n (%)	52 (34)	10 (20)	20 (38)	22 (47)
Length of stay in hospital (days), median (IQR)	5 (2,10)	4 (2,8)	6 (4,11)	6 (1,11)
Length of stay in ICU or HDU (days), median (IQR)	1.5 (1,3)	1 (0,2)	2 (1,4)	1 (1,3)
Non-operative treatment				
*Observation alone, n (%)*	108 (72)^a^	47 (94)	38 (72)	22 (47)
*Angiography with or without embolization, n (%)*	20 (13)	2 (4)	7 (13)	11 (23)
Operative treatment				
*Splenectomy/spleen-preserving surgery, n/n (%)*	20/3 (15)	0/1(2)	7/1 (15)	13/1 (30)

*OIS* Organ Injury Scale, *ISS* Injury Severity Score, *n* number of patients, *RBC* Red blood cells, *IQR* Inter Quartile Range, *ICU* Intensive Care Unit, *HDU* High Dependency Unit, ^a^unknown OIS for 1 patient

Among the 151 patients, a total of 21 are registered as transferred to the regional trauma center, and thus lost to further follow-up for the study. Before transfer 12 of these patients were observed (six patients with grade 1 or 2, three patients with grade 3, three patients with grade 4 or 5), NOM with angiography with or without embolization was undertaken in four patients (all with grade 4 or 5), four patients underwent splenectomy (one patient with grade 3, three patients with grade 4 or 5) and one patient underwent spleen preserving surgery (grade 4). The anonymized design of the study only allowed collection of data until transfer, precluding data on further treatment, complications and outcome in these patients.

A total of 18 patients were coded with spleen-related complications. One splenectomized patient underwent re-laparotomy caused by postoperative bleeding. One patient was drained for pancreatic fistula after open splenectomy, one for a suspected splenic abscess after NOM including SAE, and two patients (one treated with NOM and one with OM) were drained for pleural effusions. The remaining 12 patients had minor complications without need of invasive procedures.

There is only one registered re-bleeding in 128 patients treated non-operatively, this patient bled after NOM with SAE and successfully underwent a second SAE. However, there are incomplete data for the 16 NOM patients who were transferred to the regional trauma center. Worth noticing is that among 47 patients with a grade 4/5 splenic injury, 22 patients were treated with observation alone. The observed primary success rate for NOM is 100%, but without complete data on 16 of the transferred patients.

## Discussion

We found an incidence of approximately 4 splenic injuries per 100,000 inhabitants/year in this study. To our knowledge, there are no previous reports in Europe on the incidence and treatment of splenic injury on a national level. Our data indicate that on a national level, approximately 85% of splenic injuries were treated non-operatively with a NOM success rate of 100%. NOM included SAE in 13% of patients overall, but the lack of data on the transferred patients might underestimate the angiography and splenectomy rate. Splenectomy or spleen-preserving surgery was performed in 15% of patients.

The NOM rate is high. A large study from the US with > 10,000 patients reports a NOM rate of 68% in adults. Our study also includes children, reported with an overall higher NOM rate than adults and would probably contribute to the reported NOM success rate [18].

Recent evidence supports the use of SAE in all grade 4 and 5 injuries [2–4]. Although implemented in the treatment protocol in the trauma centers, the data shows a clear potential for improvement in practice. However, despite the lower than recommended use of SAE, the success rate is high and similar to results from other studies [1, 3].

The 30 day mortality was 4%. We do not have data on the specific cause of death, and are thus unable to assess whether it was mainly related to the splenic injury. The mortality rate is lower than in a study on splenic injury from OUHU, but our study have less seriously injured patients assessed with ISS [2]. The mortality is slightly lower than reported in a general Norwegian trauma population [19, 20].

The long-lasting interest and leadership from the trauma center at OUHU has resulted in a number of publications and initiatives on a national scale contributing to the development of the trauma care in Norway [20–22]. An initiative from the northernmost hospital in Norway, Hammerfest hospital on team training has also been implemented in all Norwegian hospitals [23]. The two national white papers on trauma care support these initiatives and seek to further develop trauma care in Norwegian hospitals with requirements for the trauma team, material resources, protocols and checklists, transfer criteria, trauma registry, mortality and morbidity conferences, and training of personnel [9, 10]. One might speculate that the presented results reflect an evolving national trauma system, with focus on transfer of injured patients who require higher levels of care including specialized treatment like endovascular procedures.

There are several limitations to this study in addition to its retrospective nature and the described challenges associated with the data collection and follow-up beyond the first admitting hospital. Many of these patients have associated injuries or premorbid medical conditions [24], which might influence management decisions and outcome. Unfortunately, we lack data to elucidate such influence further. We used the diagnostic coding system (ICD 10) to identify patients and complications, which makes the study dependent on the quality of the coding by the physicians responsible for the discharge of the patients. This might affect several of the data entry points including missing data on other injuries, and complications related to the management of the splenic injury. As all hospitals delivered anonymous data, we cannot connect patient data from acute care hospitals and regional trauma centers for the 21 transferred patients. Transferred patients might have undergone further therapy at the trauma center, leading to an underestimation of the spleen related procedures performed. As a result of lack of study approval from some data protection officers, there are very few data from the Western Regional Health Trust.

To ensure complete national data for future studies, a national trauma registry is necessary. The Norwegian Trauma Registry has included patients since 1 Jan 2015. With certified registrars in all hospitals, the registry will hopefully contribute with high quality data for upcoming national studies.

## Conclusion

Approximately 4 patients with splenic injuries per 100,000 people/year are admitted to Norwegian hospitals. Most hospitals treat very few patients with splenic injuries. We found an overall 85% primary NOM rate.
